# Supplementary material for: Human cytomegalovirus induces significant structural and functional changes in terminally differentiated human cortical neurons
Source: mBio. 2023 Nov 15;14(6):e02251-23. doi: 10.1128/mbio.02251-23 (PMC10746155; doi:10.1128/mbio.02251-23)
Supplement: Supplemental legend — Legend for Movie S1. [file mbio.02251-23-s0001.docx]

Adelman et al., 2023

Supplemental legends

**MOVIE 1 TB40/E-eGFP infection drives syncytia formation in forebrain neuron cultures***.* 3D render of syncytial structure using z stacks of 0.430 µm thickness. Blue = Hoechst 33342; Green = viral eGFP; Red = TUJ1; Magenta = Ki67.
